# The riddle of multinucleated “floret-like” giant cells and their detection in an extensive gluteal neurofibroma: a case report

**DOI:** 10.1186/1752-1947-7-189

**Published:** 2013-07-26

**Authors:** Katrin Stanger, Sora De Kerviler, Istvan Vajtai, Mihai Constantinescu

**Affiliations:** 1 Department of Plastic and Hand Surgery, University Hospital, Inselspital and University of Bern, Bern, Switzerland; 2 Department of Clinical Research, University of Bern, Bern, Switzerland; 3 Neuropathology Service, Institute of Pathology, University of Bern, Bern, Switzerland; 4 Department of Plastic and Hand Surgery, University Hospital of Erlangen, Krankenhausstrasse 12, Erlangen, 91054, Germany

**Keywords:** Floret-like multinucleated giant cells, Neurofibroma, Neurofibromatosis type 1

## Abstract

**Introduction:**

The neurofibromatoses are inherited tumor predisposition syndromes involving two major clinical phenotypes: neurofibromatosis type 1 (von Recklinghausen's disease) is linked to chromosome 17q, and tends to occur seven times more frequently than neurofibromatosis type 2. Neurofibromatosis type 1 entails a distinctive cutaneous manifestation prevailed upon by benign neurofibromas, which may vary in size, number and distribution. On the histological level, neurofibromas are composed of an admixture of neurilemmal cells, including Schwann cells, fibroblasts, and – to a lesser extent – perineurial cells.

**Case presentation:**

The case of a 39-year-old Caucasian man with a voluminous recurrent neurofibroma of 27×15cm extending from the left gluteal region to thoraco-lumbar levels Th6 through L4 is reported. Within the soft tissue tumor a pseudocyst of 7.3×9.3cm was found preoperatively.

**Conclusion:**

Histopathological study of the excised mass was conspicuous for revealing a large number of multinucleated floret-like giant cells within an otherwise classical soft tissue neurofibroma.

Previous reports on neurofibromas with multinucleated floret-like giant cells are distinctly scant. Available evidence from the literature does not suggest any consistent correlation of multinucleated floret-like giant cells in neurofibromas with gender, age, traumatic antecedents, size of the lesion, recurrence, or malignant transformation. Furthermore, the presence of such cells may not be specific for neurofibromatosis type 1, as they occasionally are encountered in some unrelated mesenchymal neoplasms as well.

## Introduction

Neurofibromatosis type 1 (NF1) is a dysgenetic complex which is due to a loss of function of the tumor-suppressor gene product neurofibromin located on chromosome 17q [1]. Mutations therefor exert a pleiotropic effect on derivatives of the neural crest including embryonic misplacement and deregulated proliferation of Schwann cells, perineural cells, and melanocytes [2]. The condition is autosomal dominant with variable penetrance and exhibits a high degree of *de novo* mutations [3]. NF1 exhibits a conspicuous cutaneous phenotype including benign neurofibromas, hyperpigmented macules ("café au lait" spots), axillary and inguinal freckling, as well as pigmented hamartomas of the iris (Lisch nodules), and distinctive osseous lesions [4,5]. Variable in number and distribution, neurofibromas are painless, slow-growing tumors. Their transformation into malignant peripheral nerve sheath tumors will occur in approximately 7 to 13% of the affected individuals [6,7]. Most of these malignant tumors tend to evolve from plexiform lesions, males being at a higher risk for such evolution [5]. Early diagnosis based on the clinical phenotype, genetic counseling, treatment of symptoms, and appropriate surgeries are the tools available for management of patients with NF1 [3]. Surgical procedures generally involve resection, combined with reconstructive strategies aimed at functional and aesthetic improvement [8]. Although the majority of neurofibromas can be excised completely by a subcutaneous approach, and closed primarily, surgery of extensive neurofibromas (massive soft tissue neurofibromas) can be challenging due to their high vascularity [3,8-10].

Although the histological identification of the NF1-associated neurofibromas is rather straightforward in practical terms, these tumors are notorious for exhibiting microscopic details that are apt to confound correct morphological diagnosis in an individual. The presence of so-called floret-like multinucleated giant cells (FMGCs) is one such detail [11].

We describe an intriguing case of an extensive lumbar and gluteal neurofibroma with FMGCs in a patient with NF1 and review the literature to investigate the association of FMGCs with NF1.

## Case presentation

**Figure 1:**
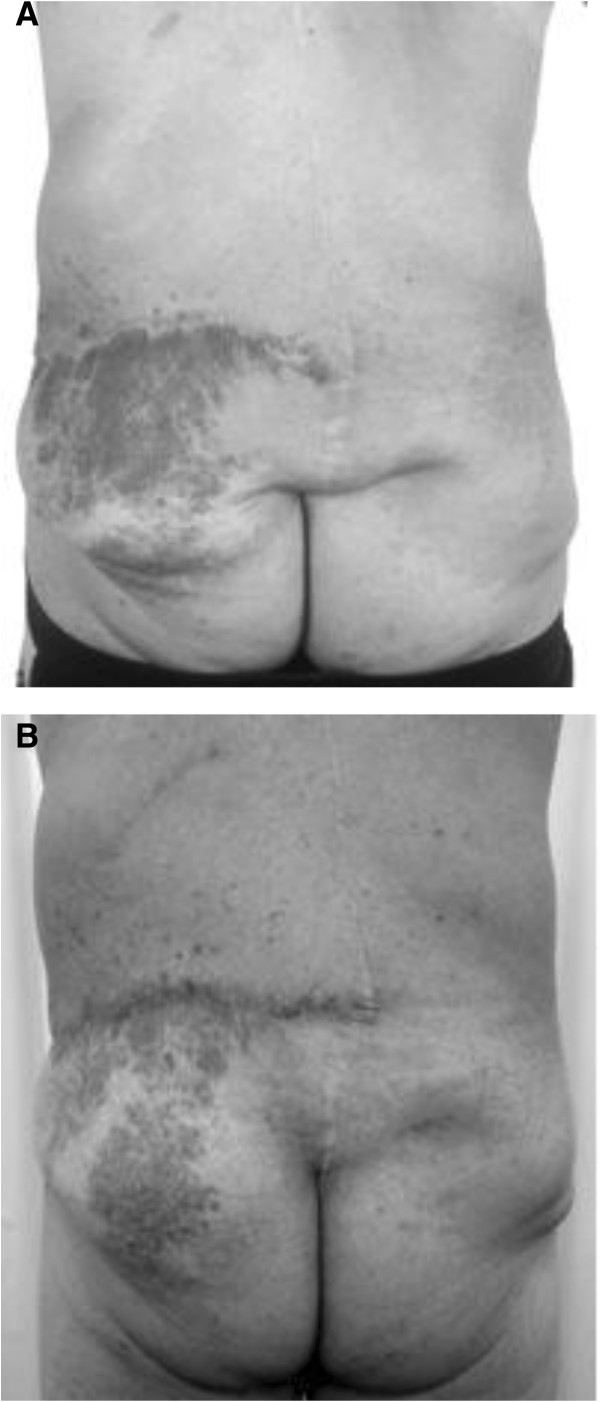
**A: Preoperative findings of the gluteal soft tissue tumor. B:** Postoperative results at 7 weeks.

A 39-year-old man of Caucasian descent with a positive family history for NF1 disease had been diagnosed with NF1 at the age of 10 years. Two years later he underwent resection of spinal and radicular neurofibromas with stabilization of the vertebral column. He presented at our out-patient service 27 years later with a recurrent soft tissue mass involving the left gluteal region along with paravertebral extension to Th6 through L4. The paravertebral and gluteal finding on the left side was a soft tissue tumor measuring 27×15cm, whereas the paravertebral tumor on the right side measured 10×13cm. The gluteal mass entailed ptosis and bulging (Figure 1A). Within the paravertebral moiety, a cyst-like fluctuation of 8×8cm was palpable. No pain, swelling or eczema was noted but enlargement of the paravertebral soft tissue tumor could be elicited by pressing. Furthermore, subcutaneous papulonodular indurations were palpable. The patient was heavily impaired by the tumor while sitting or performing physical activity, and reported on being distressed by the cosmetic deformity. Pressure on the tumor provoked sharp pain. After a history of multiple resections of several smaller neurofibromas, the tumor now showed a quick progression during one month. In addition, he developed multiple "café au lait" spots on his back, and bilateral gluteal freckling as well as multiple small neurofibromas on his trunk and both thighs. On his left forehead, a neurofibroma of 3×1.5cm was present. Lisch nodules without retinopathy were observed in both eyes.

**Figure 2:**
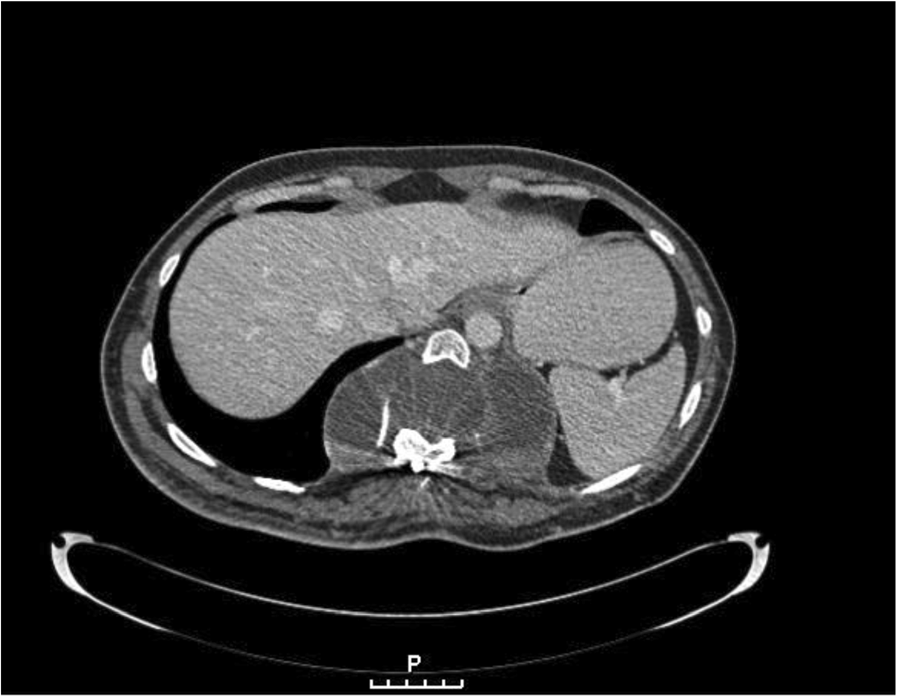
Fluid-filled vertebral cystic cavity of 8.3×20.3cm representing a meningocele.

**Figure 3:**
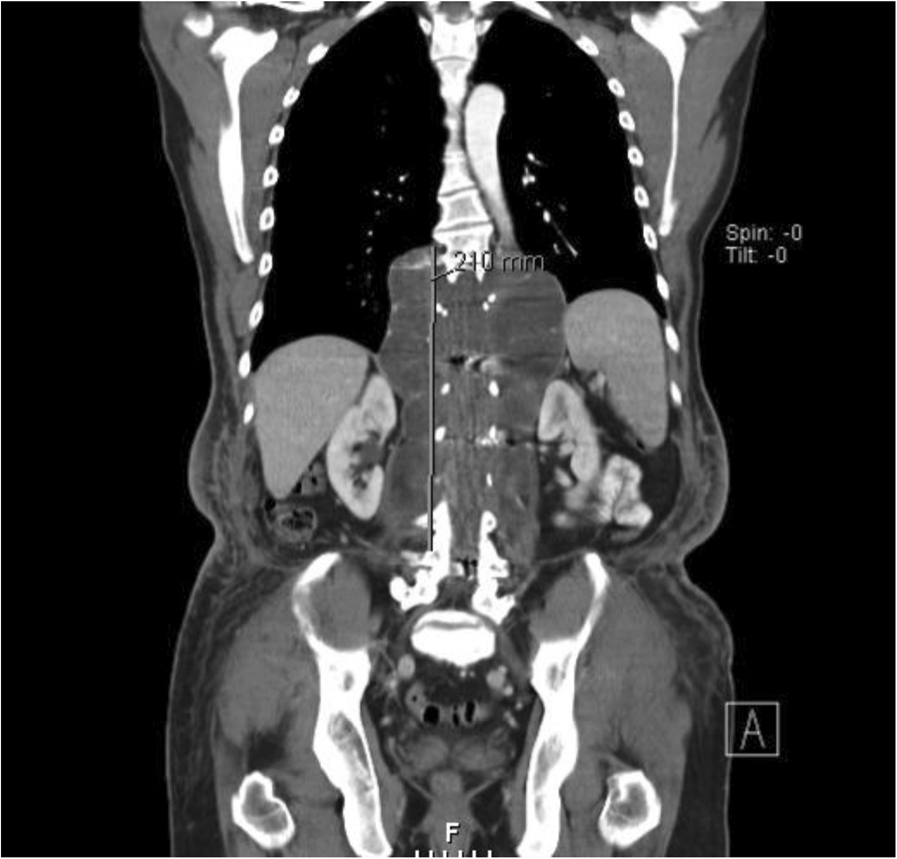
Fluid-filled vertebral cystic cavity of 8.3×20.3cm representing a meningocele.

An abdominal-pelvic computed tomography scan revealed an extensive mass lesion displacing the soft tissue of the left gluteal region and a fluid-filled vertebral cystic cavity of 8.3×20.3cm (Figures 2 and 3). Although no subcutaneous connection between the paravertebral and gluteal findings was demonstrated, the consulting neurosurgeon advised against the resection of what was interpreted to represent a meningocele.

The patient underwent wide partial excision (20×12cm) of the neurofibroma under general anesthesia. Because of the presence of irregular frail and plexus-like veins, subtle hemostasis was mandatory throughout the procedure. Direct wound closure could be achieved by advancement and rotation of the adjacent skin. Seven weeks following surgery, there was significant reduction of the left paravertebral and gluteal bulging, and an improved symmetry of the lumbar and gluteal regions (Figure 1B). The patient also reported a reduction in the localized pain he had experienced prior to resection and remained satisfied at 1 year follow-up, with no recurrence of the neurofibromatous mass.

Following overnight fixation in 10% buffered formalin, the resection specimen was routinely processed to paraffin, and 3μm-thick slides were stained with hematoxylin and eosin for microscopic study. Immunohistochemistry was performed with the following panel of antibodies: vimentin (clone Vim 3B4; Dako, Glostrup, Denmark), CD34 (clone QBend/10; Dako), S100 protein (polyclonal; Dako), smooth muscle actin (clone 1A4; Sigma, St. Louis, MO, United States of America), epithelial membrane antigen (EMA; clone E29; Dako), glucose transporter 1 (GLUT-1; polyclonal; Abcam, Cambridge, United Kingdom), according to established protocols in our laboratory. Slides were developed with polymer-bound horseradish peroxidase (Envision+; Dako) and 3,3'-diaminobenzidine as chromogen.

**Figure 4:**
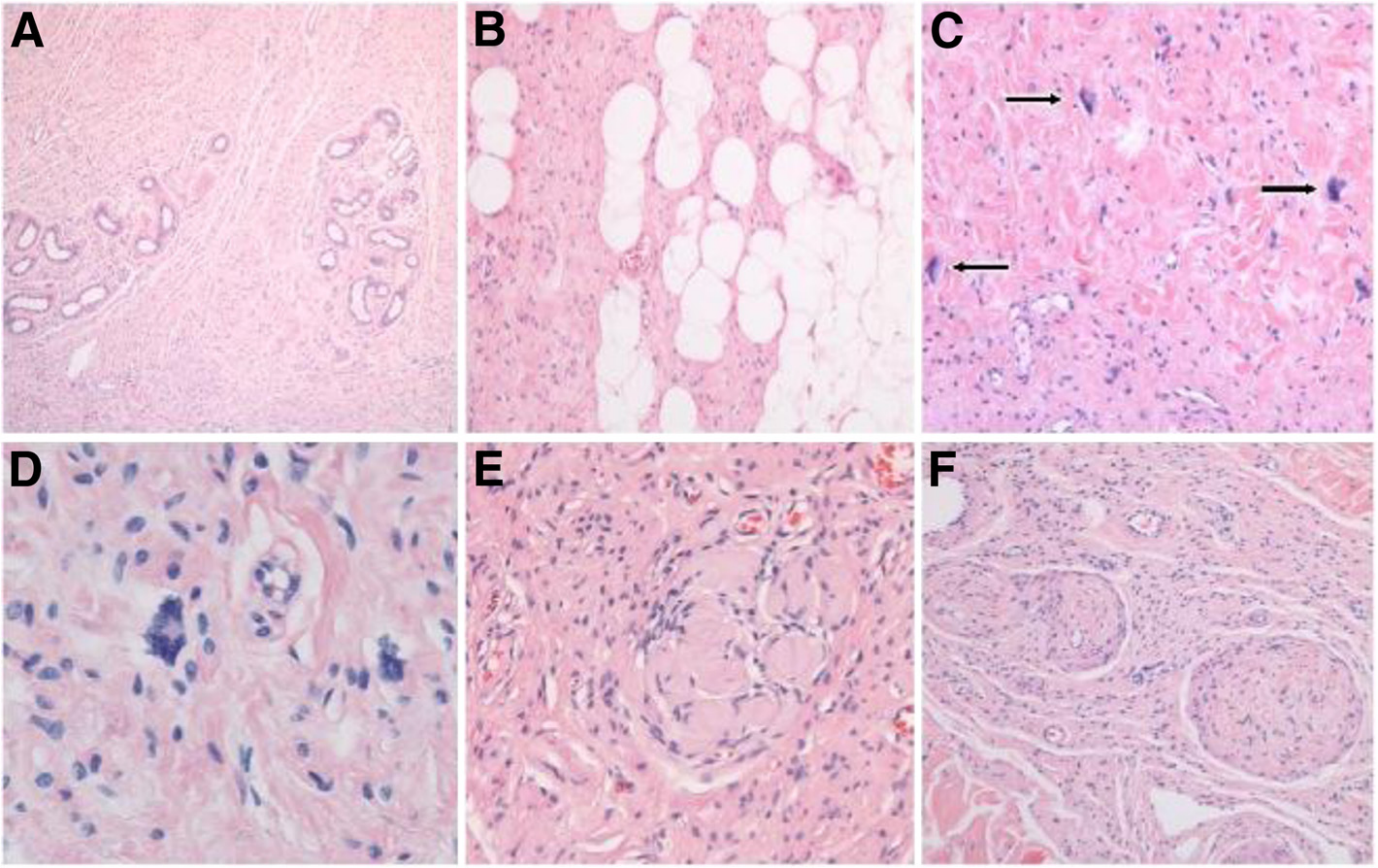
**Microscopic aspect of neurofibroma with floret-like cells. A** Overview to show diffuse infiltration of reticular dermis by neurofibroma, wherein cutaneous appendages (eccrine glands) appear permeated rather than dislocated by tumor cells. **B** Along the border to subcutaneous fat, neurofibroma cells tend to gradually merge with local adipocytes. **C** The cellular monotony of underlying neurofibroma is focally disrupted by large multinucleated cells (floret-like cells; arrows). These were felt to occur in a haphazard manner irrespective of local variation in tumor architecture. **D** High magnification view to show cytologic detail of two floret-like giant cells. Peripheral crowding of multiple nuclei along jagged cytoplasmic borders is appreciated in floret-like cell on center left. **E** Meissnerian-like differentiation is particularly frequent in – if not characteristic of – syndrome-associated neurofibromas. **F** An occasional tortuous peripheral nerve fascicle expanded by endoneurial tumor cells is felt to represent an elementary form of plexiform neurofibroma. All microphotographs represent slides stained by hematoxylin and eosin. Original magnifications: **A** – ×40; **B**, **C**, **F** – ×100; **D** – ×400; **E** – ×200.

**Figure 5:**
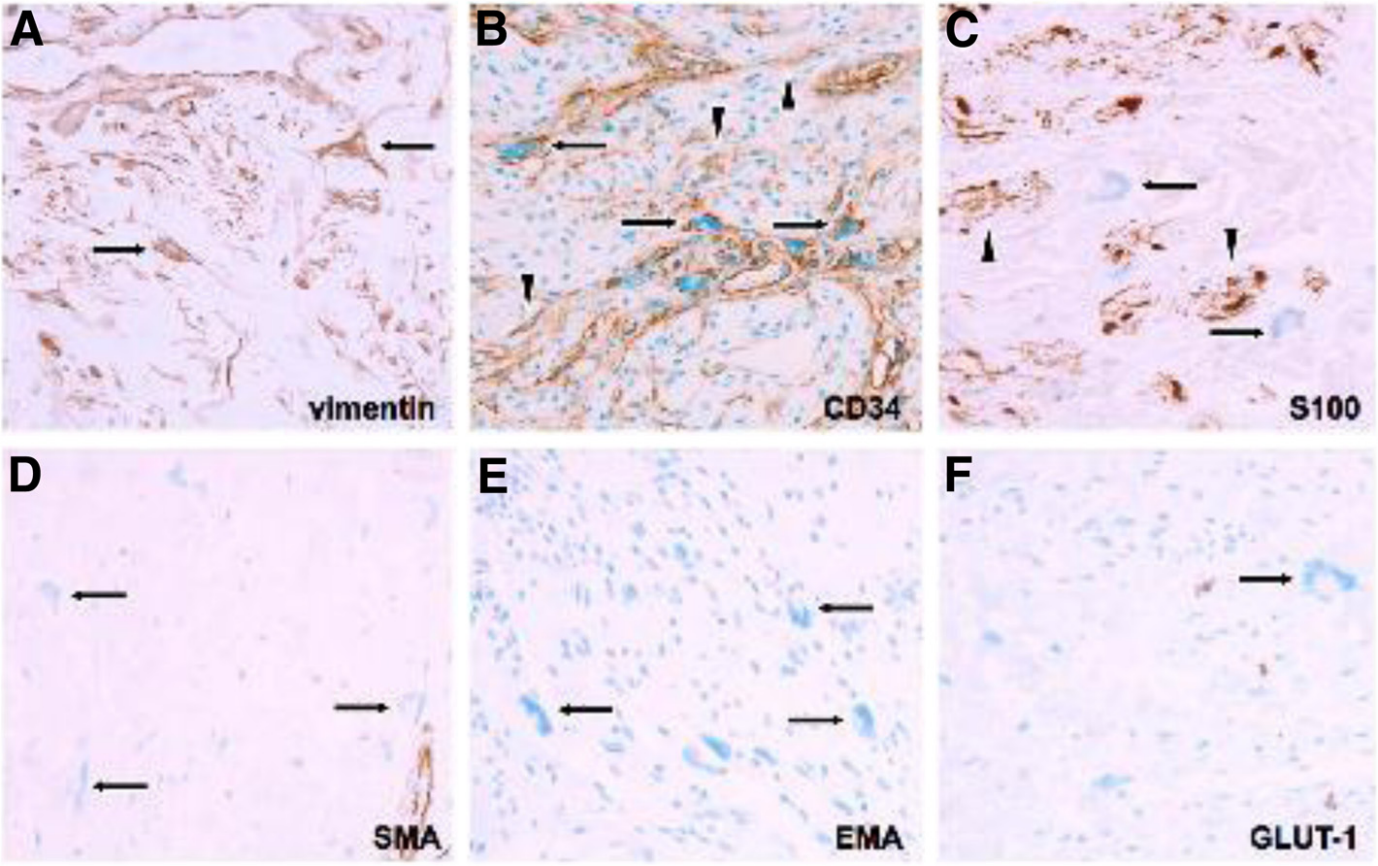
**Immunophenotype of floret-like giant cells within neurofibromatosis type 1-associated cutaneous neurofibroma. (A)** Most floret-like giant cells (arrows) exhibited intense cytoplasmic staining with the generic mesenchymal intermediate filament protein vimentin, and **(B)** shared immunoreactivity for the myofibroblastic marker CD34 (arrows) with gracile endoneurial-type fibroblasts (arrowheads) that tend to ubiquitously occur within neurofibromas. **(C)** Whereas the main population of neurofibroma tumor cells is positive for S100 protein (arrowheads), floret-like giant cells (arrows) are not. Further, consistently negative reactions included **(D)** smooth muscular actin (arrows), as well as the perineurial markers **(E)** EMA (epithelial membrane antigen) (arrows), and **(F)** GLUT-1 (glucose transporter 1) (arrow). Original magnification: **(A)** through **(F)** ×200.

Microscopic examination of the surgical specimen showed a plaque-like proliferation of spindle cells with wavy bipolar cytoplasm and mostly cigar-shaped nuclei, diffusely involving the entire breadth of the dermis (Figure 4A), and focally encroaching upon the subcutaneous fat (Figure 4B). Although there was neither significant nuclear atypia nor any diagnostically relevant mitotic activity, the tumor was conspicuous for including several aggregates of multinucleated giant cells with star-shaped cytoplasmatic projections corresponding to floret-like cells (Figure 4C, D). Furthermore, some areas of Meissner-like differentiation were seen (Figure 4D). Although an occasional peripheral nerve fascicle was felt to contain neurofibroma cells within the endoneurium (Figure 4E), no *bona fide* plexiform component was detected. The immunophenotype of the FMGCs included positivity for vimentin and CD34 (Figure 5A, B); whereas no staining was observed for S100 protein, SMA, EMA, and GLUT-1, respectively.

## Discussion

The etiology of FMGCs is unknown [11,12]. We are aware of only four previous case reports on FMGCs in neurofibromas arising in the setting of NF1 [5,13-15], as well as a recently published single retrospective clinicopathologic study of 22 patients with NF1 and FMGCs [11]. Magro *et al*. raise the question whether this phenomenon is merely an uncommon incidental finding or if it is pathognomonic for NF1 [11,12,14]. These authors suggest that FMGCs in an otherwise typical neurofibroma may not be indicators of NF1, but more probably represent reactive change of the indigenous dermal or endoneurial fibroblasts or dendritic cells in response to some unknown microenvironmental stimulus [11]. In their series of 94 neurofibromas, only 23% of the cases contained FMGCs, in 17 of these cases FMGCs cells were only identified after meticulous search. Only in 5% of the series were FMGCs found to be numerous and readily identifiable. With regard to clinical features, no correlation was found between FMGCs and gender (p=0.59) or age (<45 versus ≥45; p=0.43) [11].

Interestingly, FMGCs have also been detected in other tissues, including breast tissue in gynecomastia [13,16]. They were further described in uncommon soft tissue tumors including pleomorphic lipoma, giant cell collagenoma, giant cell fibroblastoma and giant cell angiofibroma, pseudoangiomatous stromal hyperplasia of the male breast in NF1, as well as in a breast hamartoma from a 6-year-old boy with NF1 and in a 50-year-old woman without any signs of NF1 [12,14,16,17].

Swick [15] concluded that FMGCs in neurofibromas should not be misinterpreted as harbingers of malignant transformation. Instead, they may represent a subpopulation of non–Schwannian S100 protein^-^ and CD34^+^ positive cells similar to CD34-positive endoneurial dendritic cells found in normal nerves and neurofibromas. Negativity of these cells for S100 protein combined with the absence of either cytologic atypia or mitoses assist in ruling out actual malignant transformation. FMGCs are also seen in other soft tissue tumors “not” associated with NF1: the presence of FMGCs in these neoplasms support the hypothesis that FMGCs are not specific to neurofibromas or to hyperplastic lesions associated with NF1 but, instead, represent non-neoplastic supporting cells in some tumors and true neoplastic cells in other tumors [15].

Shaktawat and Golka recommend these cells to be interpreted carefully, keeping in mind the rare malignant change in neurofibromas [14]. According to Shaktawat and Golka these are positive with vimentin and CD34 and negative with S100 and CD68 [14]. Taungjaruwinai and Goldberg, Swick and Magro *et al.* described in their cases the S100 negative FMGCs [5,13,15]. In the study by Shaktawat and Golka the FMGCs were also negative for the macrophage marker CD68 [14]. Multinucleated cells have also been known as part of the spectrum of “degenerative atypias” in neurofibromas.

The issue of possible gender predilection for FMGCs to appear in NF1-associated neurofibromas remains unclear as well.

In the case presented here, FMGCs were found in a man with NF1. Satter observed that such a constellation may be specific for males, pointing out that all recorded cases involved male patients [5,12-15]. Conversely, 53% of the study cohort in the series by Magro *et al.* was female [11]. It is not clear, however, to what respective extent male and female patients were represented among those 8% of patients in whom FMGCs actually were seen [11].

In one case report, the hypothesis has been put forward that trauma may trigger the emergence of FMGCs [15]. Hypoxia and/or reparative changes were hypothesized to have contributed to the induction of FMGCs [12,15]. Therefore it remains unclear whether recurrence, cellular stress or injury play a role in the development of FMGCs [12,13,16].

Furthermore, the size of the neurofibroma might also be a stimulating factor for the appearance of FMGCs. In our case we found a neurofibroma of 27×15cm. The literature generally describes these cells in much smaller lesions. Taungjaruwinai and Goldberg describe FMGCs in neurofibromas with an average size of 0.6cm [5]. Swick *et al.* mentioned a neurofibroma inferior to 0.8cm [15]. Magro *et al.* saw a neurofibroma with 10cm diameter where they reportedly found FMGCs; whereas Shaktawat and Golka found FMGCs in a neurofibroma of 1.2 cm [11,13,14].

Finally, surgical excision or even partial lesion reduction is indicated for alleviation of symptomatic lesions and allows final histopathological examination [3,6,7].

## Conclusion

Having discussed earlier that FMGCs are not specific for NF1, and may appear also in other tissue, further studies are warranted to clarify the etiopathogenesis of FMGCs together with possible association of long-term risk of neoplastic changes in NF1 [5,11-13]. Complete or partial surgical removal of the lesion is indicated for symptom alleviation and histopathological diagnosis of the tumor [9,18].

## Consent

Written informed consent was obtained from the patient for publication of this manuscript and accompanying images. A copy of the written consent is available for review by the Editor-in-Chief of this journal.

## Competing interests

The authors declare no conflict of interest with respect to the contents of this paper.

## Authors’ contributions

All authors have contributed significantly to the manuscript. KS: Literature search, manuscript writing and submission, surgical procedure. SDK: Manuscript review and corrections, literature review. IV: Histopathological expertise, literature review, documentation, manuscript corrections. MC: Surgical procedure, patient treatment, manuscript concept and corrections. KS investigated the case and the case history as well as the actual state of the literature. IV performed the histological examination of the tissue and MC was a major contributor in writing the manuscript. All authors read and approved the final manuscript.

## References

[B1] Viskochil D Genetics of neurofibromatosis 1 and the NF1 gene J Child Neurol 2002 17 56 10.1177/088307380201700804 12403554

[B2] Ball N Kho G Melanocytic nevi are associated with neurofibromas in neurofibromatosis type 1, but not sporadic neurofibromas: a study of 226 cases J Cutan Pathol 2005 32 523 10.1111/j.0303-6987.2005.00376.x 16115049

[B3] Renshaw A Borsetti M Nelson R Orlando A Massive plexiform neurofibroma with associated meningo-encephalocoele and occipital bone defect presenting as a cervical mass Br J Plast Surg 2003 56 514 517 10.1016/S0007-1226(03)00151-6 12890468

[B4] Gerber P Antal A Neumann N Homey B Matuschek C Peiper M Budach W Bölke E Neurofibromatosis Eur J Med Res 2009 14 102 105 19380279 10.1186/2047-783X-14-3-102 PMC3352057

[B5] Taungjaruwinai W Goldberg L Multinucleate giant cells in neurofibromas: a clue to the diagnosis of neurofibromatosis J Cutan Pathol 2009 36 1164 1167 10.1111/j.1600-0560.2009.01249.x 19281485

[B6] Lin B Weiss L Medeiros L Neurofibroma and cellular neurofibroma with atypia: a report of 14 tumors Am J Surg Pathol 1997 21 1443 10.1097/00000478-199712000-00006 9414187

[B7] Shack R Reilley A Lynch J Neurofibromas of the head and neck South Med J 1985 78 801 804 10.1097/00007611-198507000-00008 3925566

[B8] Spira A Riccardi V Neurofibromatosis Clin Plast Surg 1987 14 2 315 325 3107867

[B9] Bloem J Van der Meulen J Neurofibromatosis in plastic surgery Br J Plast Surg 1978 31 1 50 53 10.1016/0007-1226(78)90015-2 414806

[B10] McClatchey A Neurofibromatosis Annu Rev Pathol Mech Dis 2007 2 191 216 10.1146/annurev.pathol.2.010506.091940 18039098

[B11] Magro G Amico P Vecchio G Caltabiano R Castaing M Kacerovska D Kazakoy D Michal M Multinucleated floret-like giant cells in sporadic and NF-1 associated neurofibromas: a clinicopathologic study of 94 cases Virchows Arch 2010 456 71 76 10.1007/s00428-009-0859-y 19937344

[B12] Satter E Floret-like multinucleated giant cells in a neurofibroma outside the context of neurofibromatosis type 1 Am J Dermatopathol 2009 31 724 725 10.1097/DAD.0b013e3181a58275 19701070

[B13] Magro G Scavo S Ruggieri M Floretlike multinucleated giant cells in a neurofibroma from a patient with NF-1: an unusual finding for such a tumor Virchows Arch 2002 441 525 526 10.1007/s00428-002-0669-y 12516575

[B14] Shaktawat S Golka D Floret-like multinucleated giant cells in neurofibroma Diagn Pathol 2007 2 47 10.1186/1746-1596-2-47 18067673 PMC2225390

[B15] Swick B Floret-like multinucleated giant cells in a neurofibromatosis type 1- associated neurofibroma Am J Dermatopathol 2008 30 632 634 10.1097/DAD.0b013e318174e73f 19033946

[B16] Lipper S Wilson C Copeland K Pseudogynecomastia due to neurofibromatosis – a light microscopic and ultrastructural study Hum Pathol 1981 12 755 759 10.1016/S0046-8177(81)80180-3 6793500

[B17] Agabiti S Gurrera A Amico P Vasquez E Magro G Mammary hamartoma with atypical stromal cells: a potential diagnostic dilemma Pathologica 2007 99 434 437 18416336

[B18] Prokopakis E Raissaki M Bourolias C Karatzanis A Velegrakis G Massive plexiform neurofibroma and spinal deformity presenting as dysphagia Am J Otolaryngol Head Neck Med Surg 2007 28 280 283 10.1016/j.amjoto.2006.09.012 17606049

